# Characterizing the Severity of SARS-CoV-2 Variants at a Single Pediatric Center

**DOI:** 10.3389/fmed.2022.896352

**Published:** 2022-05-23

**Authors:** Aslam Khan, Caroline Ichura, Hannah Wang, Izabela Rezende, Malaya K. Sahoo, ChunHong Huang, Daniel Solis, Mamdouh Sibai, Fumiko Yamamoto, Sindiso Nyathi, Bethel Bayrau, Benjamin A. Pinsky, A. Desiree LaBeaud

**Affiliations:** ^1^Department of Pediatrics, Division of Infectious Diseases, Stanford University, Stanford, CA, United States; ^2^Department of Pathology, Stanford University, Stanford, CA, United States; ^3^Department of Medicine, Division of Infectious Diseases and Geographic Medicine, Stanford University, Stanford, CA, United States

**Keywords:** pediatrics, SARS-CoV-2, COVID-19, severity, variants, SARS-COV-2 variants

## Abstract

Since March 2020, SARS-CoV-2 has plagued the world with COVID-19 and individuals of all ages have experienced varying symptoms of disease. Older adults were experiencing more severe disease compared to children and were prioritized by vaccination efforts. While biologic therapies and vaccinations were implemented, there were changes in public health restrictions with subsequent surges resulting in more infected children. During these surges there was a rise of different SARS-CoV-2 variants with the dominant variant initially alpha (B.1.1.7 and other Pango lineages) and epsilon (B.1.427/B.1.429) in early 2021 and a dramatic shift to delta (B.1.617.2 and other Pango lineages) by mid-summer 2021. In this study we aimed to characterize the clinical severity and host factors associated with disease by SARS-CoV-2 variant and evaluate if there are differences in disease severity by circulating variant. We retrospectively included all individuals 0–25 years of age who presented to our center and had a positive SARS-CoV-2 RT-PCR, SARS-CoV-2 variant mutation testing, and documented clinical notes from 1 January 2021 through 31 December 2021. We identified 745 individuals who met inclusion criteria and found the delta variant was associated with severe/critical disease compared to the other variants studied. The results of the model showed that underlying respiratory disease and diabetes were risk factors for progression to severe disease. These insights are important when evaluating public health measures and treatment options for children as more variants arise.

## Introduction

The novel coronavirus disease 2019 (COVID-19) caused by the severe acute respiratory syndrome coronavirus 2 (SARS-CoV-2) has been associated with less severe outcomes in children in comparison to adults. Over the course of the COVID-19 pandemic, adult patients filled hospitals to capacity across the United States with children's hospitals experiencing severe cases to a lesser extent (1). As new biologic therapies and vaccinations were implemented in the setting of loosening of public health restrictions, there were surges with children becoming a larger percentage of positive cases (1). In 2021, while children were returning to school, many did not receive or did not qualify for the COVID-19 vaccination, with approval for younger ages occurring in stages through the year. Although the goal was to provide protection for a larger portion of the population, there has been a slower uptake in vaccination and decline in overall vaccination rates in the younger age groups, placing individuals at risk for infection and severe disease (2, 3).

With implementation of vaccinations alongside other SARS-CoV-2 therapeutics, there has been a rise in variants of concern for SARS-CoV-2 (4). SARS-CoV-2 is a positive-sense, single-stranded RNA virus and it is expected for RNA viruses to introduce random genomic error during replication, especially with a high replication rate that has been observed. Since the beginning of the COVID-19 pandemic, the virus has been under continuous selective pressure and evolving (5–7). This can lead to the rapid emergence and spread of viral variants with major genetic changes that have been classified as variant being monitored or variant under monitoring (VBM/VUM), variant of interest (VOI), variant of concern (VOC), and variant of high consequence (VOHC) (5).

As of November 2021, the World Health Organization (WHO) had designated five SARS-CoV-2 variants as VOCs: alpha (B.1.1.7, first detected in the UK), beta (B.1.351, first detected in South Africa), gamma (P.1, first detected in Brazil), delta (B.1.617.2, first detected in India), and omicron (B.1.1.529, first detected in South Africa) (8). In March 2021, epsilon (B.1.427 and B.1.429, first detected in California, USA) was considered a VOC, but after delta cases increased, this variant was changed to VBM in September 2021 (9).

Throughout 2021 in the United States there was a notable change in the dominant SARS-CoV-2 variant in circulation with a shift from alpha (B.1.1.7 and other Pango lineages) to delta (B.1.617.2 and other Pango lineages) by mid-year (7). Locally in Northern California Epsilon (B.1.427/B.1.429) was co-circulating with alpha (B.1.1.7) but was also replaced by delta (B.1.617.2) in co-circulation. Among all the VOCs, particularly alpha and delta were believed to carry mutations that increase the transmissibility of SARS-CoV-2 and potentially lead to more severe disease.

Studies on COVID-19 infection in children and teenagers have noted differences in infection rates, symptoms, and mortality as compared to adults (10). Children infected with SARS-CoV-2 usually have mild symptoms but can range from an asymptomatic infection to critical disease with multi-organ dysfunction (11). Additionally, various comorbidities have been associated with progression to severe disease in adults, including obesity, chronic respiratory disease, cardiovascular disease, neurological disorders, immune disorders, metabolic disease, hematologic disorders, cancer, renal disease, and gastrointestinal disease (12). Given the nature of COVID-19 disease, the majority of clinical data and research studies have occurred in adults and been applied to children. There are limited pediatric studies comparing the differences in clinical severity between variants, particularly in the Western United States. Here we conduct a single center observational study to examine the differences of SARS-CoV-2 variant infections in 2021.

## Methods

We retrospectively identified all SARS-CoV-2 RT-PCR positive individuals aged 0–25 years old that were tested through Stanford Children's Health and Stanford Healthcare at Stanford University from 1 January 2021 to 31 December 2021. Individuals had samples collected from the clinic setting, urgent care, drive-thru testing, pre-operative testing, the Emergency Room, and upon admission at the Lucile Packard Children's Hospital and Stanford Hospital. SARS-CoV-2 nucleic acid amplification testing (NAAT) and variant genotyping was performed at the Stanford Clinical Virology Laboratory. Initial respiratory SARS-CoV-2 NAAT was conducted on a variety of platforms according to manufacturer and emergency authorization instructions as previously described (13).

### Laboratory Methods

During the time frame of this study, all available specimens testing positive for SARS-CoV-2 by NAAT with reverse-transcription quantitative polymerase chain reaction (RT-qPCR) cycle threshold (Ct) <35, or transcription-mediated amplification relative light units (RLU) > 1,100 were subject to different multiplex allele-specific genotyping RT-qPCR reactions targeting spike mutations associated with known variants of concern. Due to the evolving variant landscape, individual samples may have been genotyped by one, two, or all three of the following previously-described RT-qPCR reactions: Reaction 1 targeting mutations in N501Y, E484K, L452R with internal control of wildtype N501 (13), Reaction 2 targeting mutations in del69-70, K417N, T478K with internal control of wildtype 69-70 (14), and/or Reaction 3 targeting mutation in del211-214 with internal control of *E* gene (15). Reaction 1 alone can differentiate alpha vs. epsilon vs. other, and was used exclusively from 1/1/2021-4/28/2021 when these were the dominant circulating variants. Reaction 2 in conjunction with Reaction 1 can differentiate between epsilon and delta, and was used between 4/28/2021-8/1/2021 when these two variants were co-circulating. From 8/1/2021-12/9/2021, epsilon essentially disappeared from circulation, and Reaction 1 was again used in isolation to differentiate between delta vs. alpha vs. other variants. From 12/9/2021-12/31/2021, Reaction 3 was used in conjunction with Reactions 1 and/or 2 to differentiate delta vs. omicron vs. other variants. SARS-CoV-2 whole genome sequencing was performed on 18.1% of the RT-qPCR genotyped samples as previously described to confirm genotyping assay performance and epidemiology at each of these time points (13).

We utilized the Stanford University research data repository (STARR) for the EPIC electronic medical record at both hospitals to identify individuals. A pediatric infectious physician team member also manually reviewed each chart to collect relevant clinical information. The study was approved by the Stanford University Institutional Review Board (IRB protocol 60259). Individuals were classified by the NIH/CDC criteria for acute COVID-19 disease into five categories: asymptomatic/pre-symptomatic infection, mild illness, moderate illness, severe illness, and critical illness (16). Asymptomatic/pre-symptomatic infection included individuals who tested positive for SARS-CoV-2 but did not have symptoms. Mild illness included individuals with symptoms such as fever, cough, sore throat, malaise, headache, muscle pain, nausea, vomiting, diarrhea, loss of taste, or loss of smell but who did not have shortness of breath, dyspnea, or abnormal chest imaging. Moderate illness included individuals who had evidence of lower respiratory disease during clinical assessment or imaging but oxygen saturation (SpO2) ≥ 94% on room air at sea level. Severe illness included individuals who had SpO2 <94%, a respiratory rate > 30 breaths/min, lung infiltrates > 50%, or new/increased oxygen requirement by nasal cannula or high flow nasal cannula. Critical illness included individuals who had respiratory failure (requiring non-invasive positive pressure ventilation or mechanical ventilation), septic shock, and/or multiple organ dysfunctions.

### Statistical Analysis

SAS (r) Proprietary Software 9.4, Copyright (c) 2016 by SAS Institute Inc., Cary, NC, USA was used to perform the statistical analysis of the study data. To assess the normality of distribution of continuous variables in our dataset, the Shapiro Wilk (n <2,000) test was used. Descriptive statistics for continuous variables that were normally distributed included mean ± standard deviation and median (min-max) for non-normally distributed variables. Mann Whitney-*U* test was used to compare non-normally distributed continuous variables, and independent sample *t*-test to compare normally distributed continuous variables. The Chi-Square test statistic was also used to compare independence between disease severity and variant subtype, and between disease severity and other covariates in the study. When assumptions of the Chi -square statistic used in our analysis were violated, the non-parametric equivalent Fisher's exact test was used. Univariate and multivariate proportional odds models were then used to evaluate the influence of various SARS-CoV-2 variants on disease severity. We conducted proportional odds model analysis examining factors impacting disease severity (asymptomatic, mild, moderate, and severe/critical) among our study participants. Given the low number of patients with severe and critical disease we combined these categories as all individuals in this category required oxygen support. Overall unadjusted and adjusted models did not meet the proportional odds assumption and so, we report the average effect of our models. All statistical tests were 2-sided, and *p*-values < 0.05 were considered statistically significant. Informed consent for retrospective data collection was waived as approved by the institutional review board.

## Results

Initially 2,137 SARS-CoV-2 RT-PCR positive individuals aged 0–25 years old were identified by testing through Stanford Children's Health and Stanford Healthcare at Stanford University from 1 January 2021 to 31 December 2021. Of those 1,504 had variant data and the remaining 633 without were excluded. In review of the variant data, 591 were identified to be the omicron VOC at the end of December 2021 and were excluded from this study. In review of the charts, 165 did not have any relevant clinical data and were excluded. Three individuals were identified to have multisystem inflammatory syndrome in children (MIS-C) and excluded. After meeting all the inclusion/exclusion criteria for our study, a total of 745 individuals were included for analysis as shown in Table 1. The patients' median age was 14.14 (interquartile range, 6.05 to 20.24), 50.47% of the patients were males, 48.59% were of non-Hispanic ethnicity, with only 5.64% being of African American origin. Of the 745 individuals included in our analysis, majority (68.05%) were classified as having a mild infection, with only 3.22% classified as having a severe/critical infection. The delta variant of SARS-CoV-2 was the predominant variant (57.18%) found among our study participants. With regards to comorbidities, the most reported comorbidity was underlying respiratory disease (56.11%) as shown in Tables 1, 2.

**Table 1:** Characteristics of the patients at diagnosis by outcome.

	**Asymptomatic disease**	**Mild disease**	**Moderate disease**	**Severe to critical disease**	***p*-value**	**Totals**
	***n* = 156**	***n* = 507**	***n* = 58**	***n* = 24**		***n* = 745**
Age (Median, IQR)	12.8 (5.24–18.5)	14.15 (5.92–20.6)	18.73 (13.04–22.64)	12.7 (4.92–17.2)		14.14 (6.05–20.24)
Gender						
• Female	67 (42.95)	266 (52.47)	25 (43.10)	11 (45.83)	0.136	369 (49.53)
• Male	89 (57.05)	241 (47.53)	33 (56.90)	13 (54.17)		376 (50.47)
Ethnicity						
• Hispanic/Latino	90 (57.69)	230 (45.36)	29 (50.00)	14 (58.33)	–	363 (48.72)
• Non-Hispanic	63 (40.38)	263 (51.87)	29 (50.00)	7 (29.17)		362 (48.59)
• Unknown	3 (1.92)	14 (2.76)	0 ()	3 (12.50)		20 (2.68)
Race						
• Caucasian	34 (21.79)	134 (26.43)	21 (36.21)	4 (16.67)	–	193 (25.91)
• African American	8 (5.13)	30 (5.92)	4 (6.90)	0 ()		42 (5.64)
• Asian /Pacific Islander/Native American	12 (7.69)	64 (12.62)	4 (6.90)	3 (12.5)		83 (11.14)
• Other/unknown	102 (65.38)	279 (55.03)	29 (53.45)	17 (70.83)		427 (57.36)
Body mass index						
• Obese	30 (27.78)	118 (30.89)	16 (34.78)	8 (34.78)		
• Non-Obese	78 (72.22)	264 (69.11)	30 (65.22)	15 (65.22)		
Co-Morbidities						
• Cardiovascular disease	46 (29.49)	105 (20.71)	20 (34.48)	12 (50)	0.0005	183 (24.56)
• Respiratory disease	62 (39.74)	298 (58.78)	40 (68.97)	18 (75)	<0.0001	418 (56.11)
• Renal disease	39 (25)	129 (25.44)	15 (25.86)	9 (37.50)	0.6143	192 (25.77)
• Immune system disease	6 (3.85)	23 (4.54)	1 (1.72)	4 (16.67)		34 (4.56)
• Malignancy	5 (3.21)	13 (2.56)	0 ()	4 (16.67)		22 (2.95)
• Diabetes	4 (2.56)	9 (1.78)	5 (8.62)	2 (8.33)		20 (2.68)
SARS-CoV-2 variant						
• Alpha	9 (5.77)	26 (5.13)	2 (5.26)	1 (2.63)		38 (5.10)
• Delta	54 (34.62)	319 (62.92)	35 (8.22)	18 (4.23)		426 (57.18)
• Epsilon	42 (26.92)	78 (60.94)	8 (6.25)	0 ()		128 (17.18)
• Other	51 (33.33)	84 (54.90)	13 (8.50)	5 (3.27)		153 (20.54)

**Table 2:** Characteristics of the Patients at diagnosis by exposure to different SARS-CoV-2 variants.

	**Alpha**	**Delta**	**Epsilon**	**Other**	***p*-value**	
	***n* = 38**	***n* = 426**	***n* = 128**	***n* = 153**		
Age (Median, IQR)	16.9 (6.7–21.5)	12.18 (4.8–19.7)	16.7 (10.2–21.7)	14.8 (7.4–19.8)		14.14 (6.05–20.24)
Gender						
• Female	19 (50.00)	209 (49.06)	64 (50.00)	77 (50.33)	0.9929	369 (49.53)
• Male	19 (50.00)	217 (50.94)	64 (50.00)	76 (49.67)		376 (50.47)
Ethnicity						
• Hispanic	11 (28.95)	165 (38.73)	84 (65.63)	103 (67.32)		363 (48.72)
• Non-Hispanic	25 (65.79)	245 (57.51)	43 (33.59)	49 (32.03)		362 (48.59)
• Unknown	2 (5.26)	16 (3.76)	1 (0.78)	1 (0.65)		20 (2.68)
Race						
• Caucasian	9 (23.68)	132 (30.99)	23 (17.97)	29 (18.95)		193 (25.91)
• African American	7 (18.42)	28 (6.57)	2 (1.56)	5 (3.27)		42 (5.64)
• Asian /Pacific Islander/Native American	6 (15.79)	51 (11.96)	15 (11.72)	11 (7.19)		83 (11.14)
• Other/unknown	16 (42.1)	215 (50.47)	88 (68.75)	108 (70.59)		427 (57.36)
Body mass index						
• Obese	11 (32.35)	82 (24.92)	40 (44.94)	39 (36.45)		
• Not obese	23 (67.65)	247 (75.08)	49 (55.06)	68 (63.55)		
Co-morbidities						
• Cardiovascular disease	9 (23.68)	90 (21.13)	43 (33.59)	41 (26.80)	0.0324	183 (24.56)
• Respiratory disease	24 (63.16)	222 (52.11)	78 (60.94)	94 (61.44)	0.0895	418 (56.11)
• Renal disease	19 (50.00)	97 (22.77)	37 (28.91)	39 (25.49)	0.0025	192 (25.77)
• Immune system disease	2 (5.26)	18 (4.23)	8 (6.25)	6 (3.92)	0.7686	34 (4.56)
• Malignancy	0 ()	12 (2.82)	6 (4.69)	4 (2.61)		22 (2.95)
• Diabetes	3 (7.89)	9 (2.11)	4 (3.13)	4 (2.61)		20 (2.68)
Disease severity						
• Asymptomatic	9 (23.68)	54 (12.68)	42 (32.81)	51 (33.33)		156 (20.94)
• Mild	26 (68.42)	319 (74.88)	78 (60.94)	84 (54.90)		507 (68.05)
• Moderate	2 (5.26)	35 (8.22)	8 (6.25)	13 (8.50)		58 (7.79)
• Severe	1 (2.63)	18 (4.23)	0 ()	5 (3.27)		24 (3.32)

The overall unadjusted and adjusted results of the proportional odds models examining disease severity following exposure to different SARS-CoV-2 variants were significant (*p*-value =< 0.0001). Individuals infected with SARS-CoV-2 variants other than the delta VOC were on average less likely to have severe disease as seen in both the unadjusted and adjusted models. Age was evaluated as a continuous variable and an increase in age by one unit was also likely to increase the risk of severe disease among our study participants. Unadjusted, Individuals with diabetes had a 2.8 times higher likelihood of having severe disease compared to individuals without diabetes which was statistically significant (OR = 2.84, 1.13–7.16). However, upon controlling for other independent variables in our model, there was only a 2.2 higher likelihood of severe disease among those with diabetes compared to those without diabetes (OR = 2.22, 0.83–5.96).

Our study however did find that the unadjusted model met the proportional odds assumption (*p* value = 0.84) with a 2.2-fold increase in the odds of more severe disease among individuals with underlying respiratory disease (other than COVID-19) when compared to individuals with no underlying respiratory disease (OR = 2.23, 1.63–3.06). The results from our unadjusted and adjusted proportional odds models are shown in Table 3.

**Table 3:** Unadjusted and adjusted odds ratios and confidence intervals for disease severity.

**Variable**	**Unadjusted odds ratio**	**95 % CI**	Adjusted odds ratio^*^	**95 % CI**
Variant (Ref = Delta)				
• Alpha vs. Delta	0.54	0.266–1.09	0.45	0.20–0.97
• Epsilon vs. Delta	0.354	0.233–0.538	0.25	0.15–0.43
• Other vs. Delta	0.410	0.276–0.609	0.43	0.26–0.70
• Age	1.025	1.005–1.045	1.03	1.01–1.05
Race (ref = white)				
• Asian	1.011	0.540–1.89	1.34	0.62–2.88
• Black/African American	0.83	0.41–1.69	0.98	0.39–2.46
• Native American	0.371	0.04–3.62	0.29	0.03–3.25
• Other	0.70	0.485–1.01	1.06	0.65–1.73
• Pacific Islander	1.02	0.40–2.61	1.86	0.66–5.31
• Unknown	2.1	0.746–5.94	6.94	0.61–79.53
Gender (ref = male)				
• Female	1.14	0.84–1.55	1.12	0.84–1.76
Body mass index (ref = not obese)				
• Obese	1.21	0.83–1.77	1.31	0.86–1.96
Ethnicity (ref = Hispanic/Latino)				
• Non-Hispanic	1.25	0.92–1.69	1.16	0.72–1.86
• Unknown	1.79	0.69–4.68	0.66	0.06–7.06
Co-morbidities (ref = no disease)				
• Cardiovascular disease	1.1	0.77–1.57	0.974	0.63–1.51
• Respiratory disease	2.23	1.63–3.06	1.96	1.31–2.93
• Renal disease	1.12	0.79–1.58	0.77	0.49–1.20
• Immune system disease	1.39	0.67–2.89	1.391	0.58–3.33
• Diabetes	2.84	1.13–7.16	2.22	0.83–5.96
• Malignancy	1.33	0.54–3.29	1.06	0.36–3.12

* *Adjusted for all other independent variables in the model*.

## Discussion

In this study we found few severe and critical COVID-19 cases in younger individuals, which is consistent with the available epidemiologic data (11). Delta (B.1.617.2 and sublineages) was the most prevalent variant and was associated with more severe/critical disease compared to alpha, epsilon, and others, which correlates with COVID-NET reporting of increased hospitalizations in the summer of 2021 (11). Our study captured individuals 0–25 years of age from the surrounding greater San Francisco Bay Area with representation from six different counties (San Mateo, San Francisco, Santa Clara, Alameda, Santa Cruz, and Contra Costa). To our knowledge, this was one of the first studies to compare the severity among variants in pediatric patients in the Western United States.

**Figure 1:**
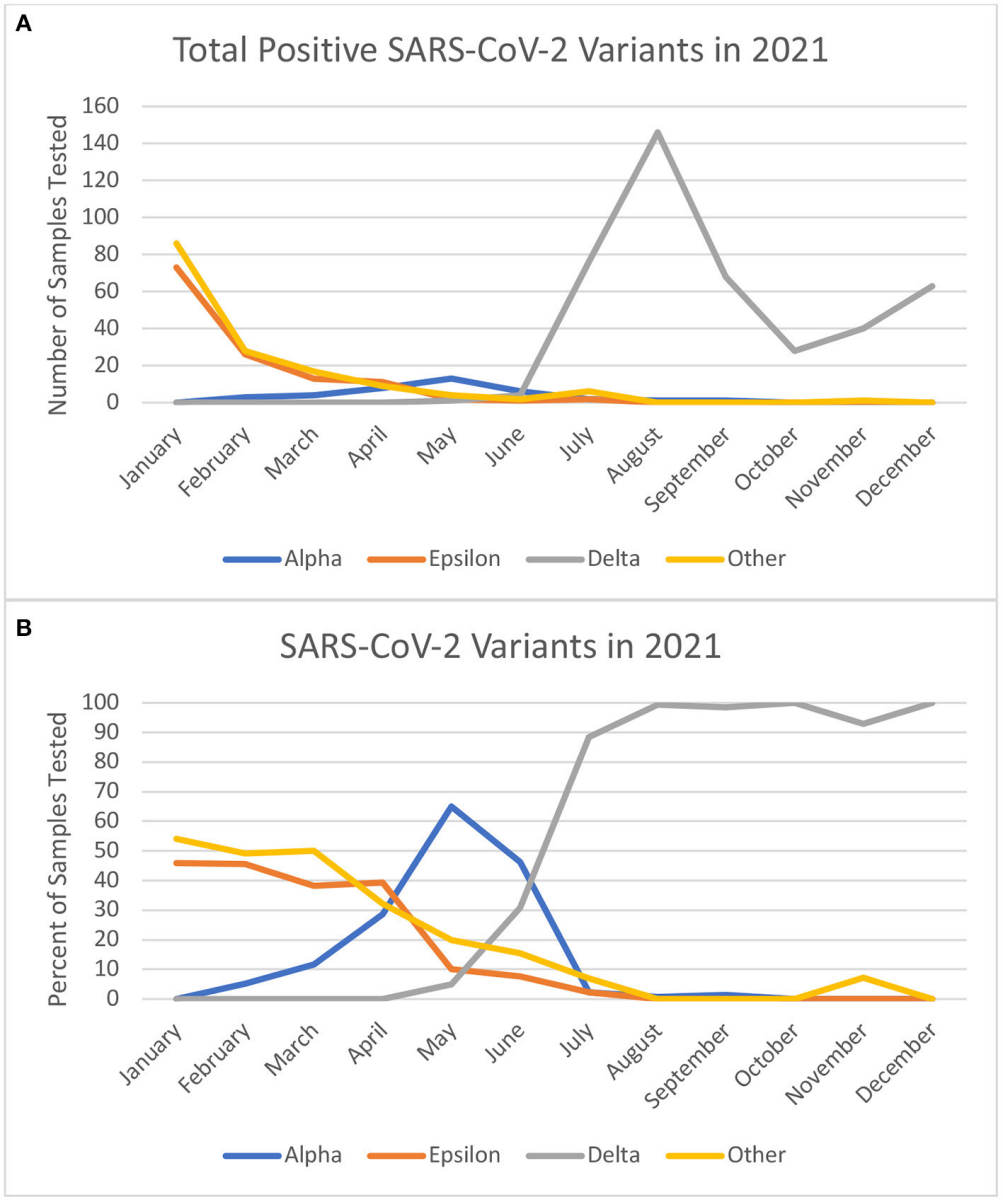
**(A)** Shows the absolute number of samples tested at our center in 2021 by month and included in the study. **(B)** Shows the monthly percentages of variant types from the positive test. Of note December does not reflect the Omicron variant which was prominent in the final week of December 2021.

There are multiple hypothetical explanations for the association of the delta VOC with more severe disease. With evolutionary changes and increased mutations in the genome encoding for the receptor binding domain and N-terminal domain of the SARS-CoV-2 spike protein, there can be more evasion from the immune system and diminished neutralizing ability (17, 18). The reduction in neutralization may explain increased transmissibility of the rising variants like delta, even in the setting of vaccination. With initial immune evasion resulting in enhanced viral entry there is an increased chance for direct viral toxicity, vasculitis induced ischemic injury, thrombosis, and immune dysregulation (19). This can provoke severe respiratory disease but also result in multi-organ disease (19). Additionally, there were social and political factors like relaxation of public health measures that may have contributed to increased transmission in mid-2021 in the region. In June 2021, the state of California lifted indoor masking mandates for vaccinated individuals and there was a subsequent rise in cases in the state and across the country (20–22). Throughout this time, most children were not eligible for vaccination with the Food and Drug Administration (FDA) granting Emergency Use Authorization (EUA) for the Pfizer-BioNTech COVID-19 vaccine for individuals aged 12–15 years in May 2021 and 5–11 years on 29 October 2021. With a rise in more cases, increase in travel, relaxation of protective measures, and ineligibility for vaccination, it is understandable more children were presenting with infection and disease. As demonstrated in Figure 1, there was a peak in the total number of positive tests at the same time the delta variant dominantly emerged in circulation.

Underlying respiratory problems and diabetes were two risk factors associated with more severe disease, similar to what is reported in the literature (12). We did not find a significant association with obesity or immunocompromised status and severe disease, although they are also risk factors defined by the CDC. Although these results have consistencies with the national data, they are not likely representative of the entire burden infection as most individuals presented for testing with symptoms or a known exposure. There were likely more asymptomatic individuals not captured given they did not present for testing.

There were several limitations in this study. The individuals included were all those who had variant testing results and clinical data. Eight hundred and one individuals were excluded and could have ranged in disease severity, as the excluded group did include multiple deaths. Fortunately the demographics were likely to be similar between genotyped and non-genotyped individuals (13, 14). Given the low proportion of individuals with diabetes among individuals with severe disease, it is likely that our study underestimated the effect of diabetes among individuals diagnosed with severe COVID-19. Studies with a higher proportion of participants with diabetes diagnosed with severe COVID-19 are needed to clearly define the odds of severe disease among children diagnosed with diabetes.

As we have progressed through the pandemic, hospital systems have required routine testing regardless of symptoms for all individuals admitted to the hospital or undergoing a procedure, capturing a larger pool of individuals which would have not been identified. With the implementation of vaccinations for the pediatric population, some individuals were potentially protected from acquiring infection, developing symptoms, or progressing to severe disease, although this effect may be minimal with variable vaccine uptake. There was also no long-term follow-up for all individuals who had asymptomatic or mild infection and it is possible some may have developed new symptoms after the initial encounter. This is less likely as we are a tertiary referral pediatric center and many other individuals who tested positive did present back to our center for additional evaluation when symptoms progressed. Another limitation is development of post-acute sequelae of COVID-19 infection which is not captured with the current data. We also excluded omicron (B.1.1.529) given there was a rise in cases at the end of December 2021 with majority of cases falling outside the window for this study, resulting in an incomplete characterization for this variant.

An additional limitation is in the methodology of multiplex RT-qPCR genotyping as a way to identify and differentiate between variants of concern. While theoretically inferior in accuracy when compared to whole genome sequencing, our RT-qPCR approach allowed for genotyping of a much larger number of samples, including samples with lower viral loads that could not be successfully sequenced. Furthermore, sensitivity and specificity of these RT-qPCR reactions when compared with whole genome sequencing was >99% in the literature (13–15). All of the samples with both RT-qPCR and sequencing data in this study had concordant results, suggesting that any unidentified genotyping errors are likely rare, and would not have influenced the final results of this study.

This study highlights that the delta variant was associated more with severe disease than prior variants in children at our center and that certain vulnerable groups (with respiratory disease and diabetes) are at risk for more severe disease. Further studies can investigate the differences with newer variants as they arise.

## Data Availability Statement

The raw data supporting the conclusions of this article will be made available by the authors, without undue reservation.

## Ethics Statement

The studies involving human participants were reviewed and approved by Stanford University Institutional Review Board. Written informed consent for participation was not provided by the participants' legal guardians/next of kin because: This was a retrospective chart review and did not directly affect any of the study participants. The standard of care was achieved for all individuals for treatment in the hospital setting independent of this study. This study in no way changed patient care or require anything further of the study participants.

## Author Contributions

AK worked on conceptualization, chart review, analysis, writing, and editing. CI worked on analysis, writing, and editing. HW worked on conceptualization, data collection, writing, and editing. MKS, CH, DS, and FY worked on data collection/generation. SN worked on analysis and editing. BB worked on data generation. BP worked on conceptualization, data collection/generation, and editing. AL worked on conceptualization, analysis, and editing. All authors contributed to the article and approved the submitted version.

## Conflict of Interest

The authors declare that the research was conducted in the absence of any commercial or financial relationships that could be construed as a potential conflict of interest.

## Publisher's Note

All claims expressed in this article are solely those of the authors and do not necessarily represent those of their affiliated organizations, or those of the publisher, the editors and the reviewers. Any product that may be evaluated in this article, or claim that may be made by its manufacturer, is not guaranteed or endorsed by the publisher.
